# (4*Z*)-4-[(2*E*)-1-Hydr­oxy-3-(4-methoxy­phen­yl)prop-2-en­ylidene]-3-methyl-1-phenyl-1*H*-pyrazol-5(4*H*)-one

**DOI:** 10.1107/S1600536809046200

**Published:** 2009-11-11

**Authors:** Khizar Iqbal Malik, Munawar Ali Munawar, Misbahul Ain Khan, Sohail Nadeem, Mukhtar-ul-Hassan

**Affiliations:** a Institute of Chemistry, University of the Punjab, New Campus, Lahore, Pakistan

## Abstract

The title compound, C_20_H_18_N_2_O_4_, is a chalcone derivative of pyrazole. The pyrazole ring is inclined at a dihedral angle of 19.29 (12)° to the methoxy­phenyl ring mean plane, and by 1.19 (13)° to the phenyl ring. The mol­ecular structure is stabilized by an intra­molecular O—H⋯O hydrogen bond, making an almost planar (r.m.s. deviation = 0.0243 Å) six membered ring.

## Related literature

For the anti­microbial activity of chalcones, see: Mityurina1 *et al.* (1981 ▶). For the syntheses of chalcones, see: Konieczny *et al.* (2007 ▶). For a heterocyclic chalcone, see: Arshad *et al.* (2008 ▶). For details concerning graphset analysis, see: Bernstein *et al.* (1995 ▶). 
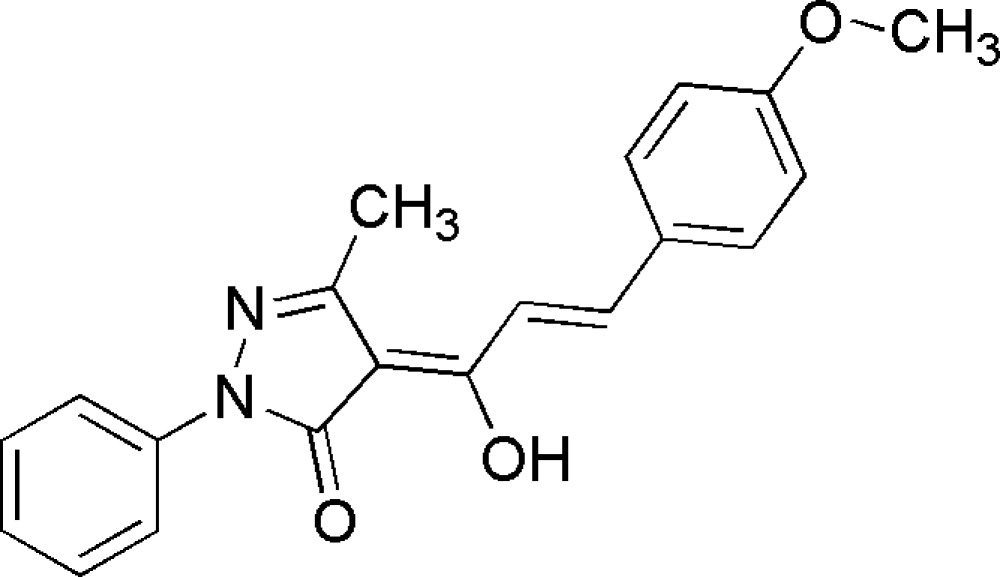


## Experimental

### 

#### Crystal data

C_20_H_18_N_2_O_3_
*M* *_r_* = 334.36
Monoclinic, 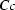

*a* = 5.0803 (2) Å
*b* = 22.7645 (9) Å
*c* = 14.5880 (6) Å
β = 97.626 (2)°
*V* = 1672.19 (12) Å^3^
*Z* = 4
Mo *K*α radiation
μ = 0.09 mm^−1^
*T* = 296 K
0.33 × 0.24 × 0.18 mm

#### Data collection

Bruker Kappa APEXII CCD diffractometer
Absorption correction: multi-scan (*SADABS*; Bruker, 2001 ▶) *T*_min_ = 0.971, *T*_max_ = 0.984
9137 measured reflections
2056 independent reflections
2628 reflections with *I* > 2σ(*I*)
*R*_int_ = 0.027

#### Refinement

*R*[*F* ^2^ > 2σ(*F* ^2^)] = 0.037
*wR*(*F* ^2^) = 0.094
*S* = 1.05
2056 reflections
229 parameters
2 restraints
H-atom parameters constrained
Δρ_max_ = 0.10 e Å^−3^
Δρ_min_ = −0.16 e Å^−3^

### 

Data collection: *APEX2* (Bruker, 2007 ▶); cell refinement: *SAINT* (Bruker, 2007 ▶); data reduction: *SAINT*; program(s) used to solve structure: *SHELXS97* (Sheldrick, 2008 ▶); program(s) used to refine structure: *SHELXL97* (Sheldrick, 2008 ▶); molecular graphics: *PLATON* (Spek, 2009 ▶); software used to prepare material for publication: *WinGX* (Farrugia, 1999 ▶) and *PLATON*.

## Supplementary Material

Crystal structure: contains datablocks I, global. DOI: 10.1107/S1600536809046200/su2151sup1.cif

Structure factors: contains datablocks I. DOI: 10.1107/S1600536809046200/su2151Isup2.hkl

Additional supplementary materials:  crystallographic information; 3D view; checkCIF report

## Figures and Tables

**Table 1:** Hydrogen-bond geometry (Å, °)

*D*—H⋯*A*	*D*—H	H⋯*A*	*D*⋯*A*	*D*—H⋯*A*
O2—H2*O*⋯O1	0.82	1.77	2.529 (3)	153

**Fig. 1.**
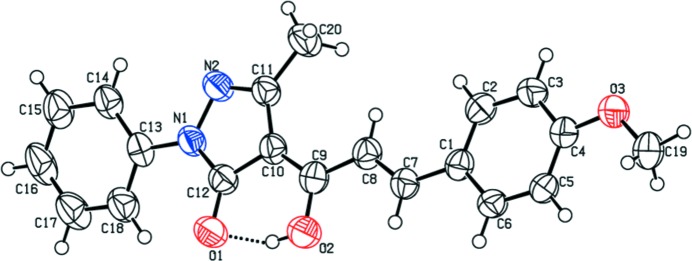
The molecular structure of the title compound, with thermal ellipsoids drawn at the 50% probability level. The intramolecular O-H···O hydrogen bond is shown as a dashed line.

## supplementary crystallographic information

### Comment

Pyranopyrazole derivatives have been reported as being antimicrobial agents (Mityurina1 *et al.*, 1981). The title compound is a heterocyclic chalcone (Arshad *et al.*, 2008), and was synthesized as we are interested in the synthesis of pyranopyrazole derivatives.

The molecular structure of the title compound is illustrated in Fig. 1, and the geometrical parameters are available in the archived CIF. The title molecule, besides the methoxy phenyl ring A (C13-C18) attached to the pyrazole ring, is almost planar. The dihedral angle between the pyrazole ring B (N1/N2/C10-C12) and phenyl ring A is 19.29 (12) °. Phenyl ring C (C1-C6) lies in the plane of the pyrazole ring B, with a dihedral angle of 1.19 (13)°. There is an intramolecular O-H···O hydrogen bond stabilizing the molecule (Fig. 1 and Table 1). It forms a six membered ring motif which can be described as *S*(6) (Bernstein, *et al.*, 1995).

### Experimental

The title compound was prepared according to the literature method (Konieczny *et al.*, 2007). 1 mmol (0.216 g) of 3-methyl-1-phenyl-acetyl-5-hydroxy pyrazole and 1.5 mmol (0.204 g) of 4-methoxybenzaldehyde was added to the mixture of 2 ml of glacial acetic acid and 0.2 ml of concentrated sulfuric acid and heated at 353-358 K for 9 h with stirring. The progress of the reaction was followed by TLC. On completion, the mixture was added to ice cold water. The precipitate obtained was filtered off, washed with methanol and purified by column chromatography using n-hexane:ethyl acetate(3:2). Red needle-like crystals, suitable for X-ray analysis, were obtained by slow evaporation of a solution in chloroform at r.t.

### Refinement

In the final cycles of refinement, in the absence of significant anomalous scattering effects, 672 Friedel pairs were merged and Δf " set to zero. The H-atoms were included in calculated positions and treated as riding: C—H = 0.93 Å for aromatic, C–H = 0.96 Å for CH_3_ and O—H = 0.82 Å, with *U*_iso_(H) = k × *U*_eq_(parent C- or O-atom), where k = 1.2 for aromatic H-atoms and 1.5*U*_eq_(parent O-atom, and methyl C-atoms).

### Crystal data

**Table:**

C_20_H_18_N_2_O_3_	*F*(000) = 704
*M**_r_* = 334.36	*D*_x_ = 1.328 Mg m^−^^3^
Monoclinic, *C**c*	Mo *K*α radiation, λ = 0.71073 Å
Hall symbol: C -2yc	Cell parameters from 3228 reflections
*a* = 5.0803 (2) Å	θ = 2.3–25.8°
*b* = 22.7645 (9) Å	µ = 0.09 mm^−^^1^
*c* = 14.5880 (6) Å	*T* = 296 K
β = 97.626 (2)°	Needle, red
*V* = 1672.19 (12) Å^3^	0.33 × 0.24 × 0.18 mm
*Z* = 4	

### Data collection

**Table:**

Bruker Kappa APEXII CCD diffractometer	2056 independent reflections
Radiation source: fine-focus sealed tube	2628 reflections with *I* > 2σ(*I*)
graphite	*R*_int_ = 0.027
φ and ω scans	θ_max_ = 28.3°, θ_min_ = 2.3°
Absorption correction: multi-scan (*SADABS*; Bruker, 2001)	*h* = −6→6
*T*_min_ = 0.971, *T*_max_ = 0.984	*k* = −30→30
9137 measured reflections	*l* = −18→18

### Refinement

**Table:**

Refinement on *F*^2^	Primary atom site location: structure-invariant direct methods
Least-squares matrix: full	Secondary atom site location: difference Fourier map
*R*[*F*^2^ > 2σ(*F*^2^)] = 0.037	Hydrogen site location: inferred from neighbouring sites
*wR*(*F*^2^) = 0.094	H-atom parameters constrained
*S* = 1.05	*w* = 1/[σ^2^(*F*_o_^2^) + (0.0492*P*)^2^ + 0.0958*P*] where *P* = (*F*_o_^2^ + 2*F*_c_^2^)/3
2056 reflections	(Δ/σ)_max_ < 0.001
229 parameters	Δρ_max_ = 0.10 e Å^−^^3^
2 restraints	Δρ_min_ = −0.16 e Å^−^^3^

### Special details

**Table:**

Geometry. Bond distances, angles etc. have been calculated using the rounded fractional coordinates. All su's are estimated from the variances of the (full) variance-covariance matrix. The cell esds are taken into account in the estimation of distances, angles and torsion angles
Refinement. Refinement of *F*^2^ against ALL reflections. The weighted *R*-factor *wR* and goodness of fit *S* are based on *F*^2^, conventional *R*-factors *R* are based on *F*, with *F* set to zero for negative *F*^2^. The threshold expression of *F*^2^ > 2σ(*F*^2^) is used only for calculating *R*-factors(gt) *etc*. and is not relevant to the choice of reflections for refinement. *R*-factors based on *F*^2^ are statistically about twice as large as those based on *F*, and *R*- factors based on ALL data will be even larger.

### Fractional atomic coordinates and isotropic or equivalent isotropic displacement parameters (Å^2^)

**Table:**

	*x*	*y*	*z*	*U*_iso_*/*U*_eq_	
O1	−0.0176 (4)	0.44681 (8)	0.51341 (14)	0.0664 (7)	
O2	0.3841 (4)	0.46097 (8)	0.63360 (16)	0.0711 (7)	
O3	1.6238 (3)	0.37711 (9)	1.04514 (13)	0.0698 (7)	
N1	−0.1696 (3)	0.34974 (9)	0.51051 (14)	0.0509 (7)	
N2	−0.0989 (4)	0.29740 (9)	0.55796 (15)	0.0553 (7)	
C1	1.0015 (4)	0.41049 (11)	0.83769 (17)	0.0485 (7)	
C2	1.0508 (5)	0.35476 (12)	0.8761 (2)	0.0582 (9)	
C3	1.2597 (5)	0.34518 (11)	0.9444 (2)	0.0596 (9)	
C4	1.4271 (4)	0.39123 (12)	0.97629 (17)	0.0525 (8)	
C5	1.3838 (4)	0.44665 (10)	0.93893 (17)	0.0493 (8)	
C6	1.1739 (4)	0.45543 (11)	0.87062 (17)	0.0503 (8)	
C7	0.7813 (4)	0.42297 (12)	0.76621 (17)	0.0509 (8)	
C8	0.5954 (4)	0.38652 (12)	0.72645 (17)	0.0520 (8)	
C9	0.3848 (4)	0.40523 (12)	0.65663 (17)	0.0520 (8)	
C10	0.1866 (4)	0.36961 (11)	0.61184 (16)	0.0476 (7)	
C11	0.1116 (4)	0.30908 (11)	0.61679 (17)	0.0499 (8)	
C12	−0.0042 (4)	0.39408 (11)	0.54131 (16)	0.0508 (8)	
C13	−0.3813 (4)	0.34899 (10)	0.43652 (17)	0.0495 (8)	
C14	−0.5624 (5)	0.30347 (11)	0.43192 (19)	0.0546 (8)	
C15	−0.7691 (5)	0.30167 (13)	0.3600 (2)	0.0689 (10)	
C16	−0.7933 (6)	0.34472 (14)	0.2929 (2)	0.0789 (11)	
C17	−0.6134 (6)	0.38955 (15)	0.2983 (2)	0.0745 (11)	
C18	−0.4058 (5)	0.39282 (13)	0.3700 (2)	0.0646 (10)	
C19	1.8035 (5)	0.42227 (14)	1.0791 (2)	0.0716 (10)	
C20	0.2392 (5)	0.26103 (12)	0.6752 (2)	0.0690 (10)	
H2	0.94040	0.32360	0.85510	0.0700*	
H2O	0.25380	0.46790	0.59560	0.1070*	
H3	1.28940	0.30780	0.96930	0.0720*	
H5	1.49530	0.47760	0.95970	0.0590*	
H6	1.14600	0.49280	0.84550	0.0600*	
H7	0.76850	0.46160	0.74540	0.0610*	
H8	0.60180	0.34730	0.74450	0.0620*	
H14	−0.54550	0.27420	0.47690	0.0650*	
H15	−0.89240	0.27130	0.35690	0.0830*	
H16	−0.93130	0.34320	0.24420	0.0950*	
H17	−0.63050	0.41850	0.25290	0.0890*	
H18	−0.28550	0.42380	0.37350	0.0770*	
H19A	1.87980	0.43950	1.02860	0.1080*	
H19B	1.94210	0.40600	1.12290	0.1080*	
H19C	1.71030	0.45190	1.10880	0.1080*	
H20A	0.14180	0.22520	0.66150	0.1040*	
H20B	0.23990	0.27080	0.73920	0.1040*	
H20C	0.41850	0.25590	0.66260	0.1040*	

### Atomic displacement parameters (Å^2^)

**Table:**

	*U*^11^	*U*^22^	*U*^33^	*U*^12^	*U*^13^	*U*^23^
O1	0.0690 (11)	0.0494 (10)	0.0744 (13)	−0.0022 (9)	−0.0138 (9)	0.0081 (9)
O2	0.0694 (11)	0.0573 (11)	0.0793 (14)	−0.0111 (9)	−0.0170 (10)	0.0090 (10)
O3	0.0580 (10)	0.0666 (12)	0.0776 (13)	−0.0102 (9)	−0.0174 (9)	0.0148 (10)
N1	0.0482 (10)	0.0453 (12)	0.0552 (13)	0.0029 (8)	−0.0083 (9)	0.0015 (9)
N2	0.0576 (11)	0.0460 (11)	0.0581 (13)	0.0010 (9)	−0.0075 (10)	0.0045 (10)
C1	0.0451 (11)	0.0504 (13)	0.0490 (14)	−0.0043 (10)	0.0021 (10)	−0.0069 (11)
C2	0.0532 (13)	0.0502 (14)	0.0680 (17)	−0.0098 (11)	−0.0042 (12)	−0.0042 (13)
C3	0.0554 (13)	0.0447 (13)	0.0749 (18)	−0.0041 (11)	−0.0053 (13)	0.0066 (13)
C4	0.0452 (12)	0.0557 (15)	0.0550 (15)	−0.0021 (11)	0.0012 (11)	0.0037 (12)
C5	0.0464 (12)	0.0465 (13)	0.0527 (14)	−0.0076 (10)	−0.0014 (10)	−0.0036 (11)
C6	0.0510 (11)	0.0453 (13)	0.0529 (15)	−0.0055 (10)	0.0005 (11)	0.0022 (11)
C7	0.0465 (13)	0.0520 (14)	0.0521 (15)	−0.0015 (10)	−0.0010 (10)	0.0000 (12)
C8	0.0477 (12)	0.0537 (15)	0.0529 (15)	−0.0022 (10)	0.0007 (11)	−0.0045 (11)
C9	0.0479 (12)	0.0563 (15)	0.0510 (15)	−0.0013 (10)	0.0038 (11)	−0.0039 (11)
C10	0.0428 (11)	0.0498 (13)	0.0483 (14)	0.0020 (10)	−0.0012 (10)	−0.0018 (11)
C11	0.0490 (12)	0.0469 (13)	0.0511 (14)	0.0014 (10)	−0.0038 (10)	−0.0008 (11)
C12	0.0505 (12)	0.0450 (14)	0.0550 (16)	0.0021 (10)	0.0003 (11)	0.0002 (11)
C13	0.0448 (12)	0.0528 (14)	0.0484 (14)	0.0112 (10)	−0.0027 (10)	−0.0070 (11)
C14	0.0518 (13)	0.0520 (14)	0.0560 (15)	0.0073 (11)	−0.0073 (11)	−0.0062 (12)
C15	0.0585 (14)	0.0662 (17)	0.075 (2)	0.0023 (12)	−0.0167 (13)	−0.0126 (15)
C16	0.0740 (18)	0.077 (2)	0.074 (2)	0.0172 (16)	−0.0334 (16)	−0.0075 (17)
C17	0.0805 (18)	0.072 (2)	0.0643 (19)	0.0138 (16)	−0.0156 (15)	0.0063 (15)
C18	0.0625 (16)	0.0652 (18)	0.0615 (17)	0.0041 (13)	−0.0082 (13)	0.0047 (14)
C19	0.0574 (14)	0.081 (2)	0.0697 (18)	−0.0097 (14)	−0.0168 (13)	0.0025 (16)
C20	0.0720 (17)	0.0543 (16)	0.0721 (18)	−0.0016 (13)	−0.0225 (14)	0.0081 (14)

### Geometric parameters (Å, °)

**Table:**

O1—C12	1.266 (3)	C13—C14	1.381 (3)
O2—C9	1.313 (3)	C14—C15	1.383 (4)
O3—C4	1.358 (3)	C15—C16	1.379 (4)
O3—C19	1.420 (3)	C16—C17	1.365 (5)
O2—H2O	0.8200	C17—C18	1.385 (4)
N1—N2	1.401 (3)	C2—H2	0.9300
N1—C13	1.419 (3)	C3—H3	0.9300
N1—C12	1.352 (3)	C5—H5	0.9300
N2—C11	1.307 (3)	C6—H6	0.9300
C1—C2	1.396 (4)	C7—H7	0.9300
C1—C7	1.453 (3)	C8—H8	0.9300
C1—C6	1.390 (3)	C14—H14	0.9300
C2—C3	1.373 (4)	C15—H15	0.9300
C3—C4	1.391 (4)	C16—H16	0.9300
C4—C5	1.380 (4)	C17—H17	0.9300
C5—C6	1.374 (3)	C18—H18	0.9300
C7—C8	1.331 (3)	C19—H19A	0.9600
C8—C9	1.440 (3)	C19—H19B	0.9600
C9—C10	1.387 (3)	C19—H19C	0.9600
C10—C11	1.434 (3)	C20—H20A	0.9600
C10—C12	1.430 (3)	C20—H20B	0.9600
C11—C20	1.482 (4)	C20—H20C	0.9600
C13—C18	1.386 (4)		
			
C4—O3—C19	117.6 (2)	C16—C17—C18	121.4 (3)
C9—O2—H2O	109.00	C13—C18—C17	118.6 (3)
N2—N1—C12	111.33 (18)	C1—C2—H2	119.00
N2—N1—C13	118.97 (19)	C3—C2—H2	119.00
C12—N1—C13	129.6 (2)	C2—C3—H3	120.00
N1—N2—C11	106.58 (19)	C4—C3—H3	120.00
C2—C1—C6	117.3 (2)	C4—C5—H5	120.00
C6—C1—C7	119.6 (2)	C6—C5—H5	120.00
C2—C1—C7	123.1 (2)	C1—C6—H6	119.00
C1—C2—C3	121.1 (2)	C5—C6—H6	119.00
C2—C3—C4	120.2 (2)	C1—C7—H7	116.00
O3—C4—C3	115.2 (2)	C8—C7—H7	116.00
O3—C4—C5	124.9 (2)	C7—C8—H8	119.00
C3—C4—C5	119.9 (2)	C9—C8—H8	119.00
C4—C5—C6	119.1 (2)	C13—C14—H14	120.00
C1—C6—C5	122.4 (2)	C15—C14—H14	120.00
C1—C7—C8	128.7 (2)	C14—C15—H15	120.00
C7—C8—C9	122.8 (2)	C16—C15—H15	120.00
O2—C9—C8	116.3 (2)	C15—C16—H16	120.00
O2—C9—C10	117.9 (2)	C17—C16—H16	120.00
C8—C9—C10	125.8 (2)	C16—C17—H17	119.00
C9—C10—C11	136.0 (2)	C18—C17—H17	119.00
C9—C10—C12	119.5 (2)	C13—C18—H18	121.00
C11—C10—C12	104.56 (19)	C17—C18—H18	121.00
N2—C11—C20	119.0 (2)	O3—C19—H19A	110.00
C10—C11—C20	129.7 (2)	O3—C19—H19B	109.00
N2—C11—C10	111.2 (2)	O3—C19—H19C	109.00
O1—C12—N1	126.6 (2)	H19A—C19—H19B	109.00
N1—C12—C10	106.3 (2)	H19A—C19—H19C	109.00
O1—C12—C10	127.1 (2)	H19B—C19—H19C	109.00
N1—C13—C14	118.6 (2)	C11—C20—H20A	109.00
C14—C13—C18	120.6 (2)	C11—C20—H20B	109.00
N1—C13—C18	120.8 (2)	C11—C20—H20C	109.00
C13—C14—C15	119.5 (2)	H20A—C20—H20B	110.00
C14—C15—C16	120.3 (3)	H20A—C20—H20C	109.00
C15—C16—C17	119.6 (3)	H20B—C20—H20C	109.00
			
C19—O3—C4—C3	178.9 (2)	C4—C5—C6—C1	0.3 (4)
C19—O3—C4—C5	−2.2 (3)	C1—C7—C8—C9	179.2 (2)
C12—N1—N2—C11	−1.0 (3)	C7—C8—C9—C10	179.4 (2)
C13—N1—N2—C11	175.09 (19)	C7—C8—C9—O2	−0.1 (4)
N2—N1—C12—C10	0.6 (2)	O2—C9—C10—C11	−178.3 (3)
C13—N1—C12—C10	−174.9 (2)	C8—C9—C10—C12	−177.7 (2)
N2—N1—C13—C14	20.3 (3)	O2—C9—C10—C12	1.8 (3)
C12—N1—C13—C14	−164.5 (2)	C8—C9—C10—C11	2.3 (4)
N2—N1—C13—C18	−159.5 (2)	C12—C10—C11—C20	178.0 (2)
C12—N1—C13—C18	15.8 (4)	C9—C10—C12—O1	−0.5 (4)
N2—N1—C12—O1	−178.9 (2)	C9—C10—C12—N1	179.9 (2)
C13—N1—C12—O1	5.5 (4)	C11—C10—C12—O1	179.5 (2)
N1—N2—C11—C20	−177.8 (2)	C11—C10—C12—N1	−0.1 (2)
N1—N2—C11—C10	0.9 (3)	C9—C10—C11—N2	179.5 (3)
C7—C1—C2—C3	−179.2 (2)	C9—C10—C11—C20	−2.0 (5)
C2—C1—C6—C5	−0.8 (4)	C12—C10—C11—N2	−0.5 (3)
C6—C1—C2—C3	0.7 (4)	N1—C13—C14—C15	−179.5 (2)
C6—C1—C7—C8	−179.5 (2)	C18—C13—C14—C15	0.2 (4)
C2—C1—C7—C8	0.4 (4)	N1—C13—C18—C17	178.9 (2)
C7—C1—C6—C5	179.2 (2)	C14—C13—C18—C17	−0.8 (4)
C1—C2—C3—C4	−0.2 (4)	C13—C14—C15—C16	0.5 (4)
C2—C3—C4—C5	−0.3 (4)	C14—C15—C16—C17	−0.7 (4)
C2—C3—C4—O3	178.7 (2)	C15—C16—C17—C18	0.0 (5)
O3—C4—C5—C6	−178.7 (2)	C16—C17—C18—C13	0.7 (4)
C3—C4—C5—C6	0.2 (3)		

### Hydrogen-bond geometry (Å, °)

**Table:**

*D*—H···*A*	*D*—H	H···*A*	*D*···*A*	*D*—H···*A*
O2—H2O···O1	0.82	1.77	2.529 (3)	153
C7—H7···O2	0.93	2.37	2.743 (3)	104
C14—H14···N2	0.93	2.47	2.792 (3)	100
C18—H18···O1	0.93	2.36	2.947 (3)	121
